# Microbiome Engineering Using Probiotic Yeast: *Saccharomyces boulardii* and the Secreted Human Lysozyme Lead to Changes in the Gut Microbiome and Metabolome of Mice

**DOI:** 10.1128/spectrum.00780-23

**Published:** 2023-07-12

**Authors:** Jungyeon Kim, Christine Atkinson, Michael J. Miller, Kyoung Heon Kim, Yong-Su Jin

**Affiliations:** a Carl R. Woese Institute for Genomic Biology, University of Illinois at Urbana-Champaign, Urbana, Illinois, USA; b Department of Food Science and Human Nutrition, University of Illinois at Urbana-Champaign, Urbana, Illinois, USA; c Department of Biotechnology, Graduate School, Korea University, Seoul, Republic of Korea; University of Michigan-Ann Arbor

**Keywords:** *Saccharomyces boulardii*, human lysozyme, metabolic engineering, metabolomics, microbiome, microbiome engineering

## Abstract

The probiotic yeast *Saccharomyces boulardii* has great potential for use as a chassis for microbiome engineering because of its high resistance to environmental stress, well-developed genetic tools, and the ability to secrete recombinant proteins in the intestine. As oral feeding of lysozyme has been reported to change the gut microbiome and fecal metabolites, we engineered *S. boulardii* to secrete human lysozyme, and investigated the changes in the microbiome and fecal metabolites in response to the administration of the engineered probiotic yeast into mice. Administration of *S. boulardii* changed the structure of the gut microbiome by promoting the growth of clostridia and increasing the diversity of strains. The human lysozyme secreted by *S. boulardii* in the intestine resulted in a unique gut microbiome structure through selective growth. In addition, the administration of probiotic yeast *S. boulardii* affected host energy metabolism and decreased blood urea and fructose levels, suggesting a mechanism of health benefits in mice.

**IMPORTANCE** Our study identified changes in the microbiome by administering wild-type *S. boulardii* in mice to healthy mice based on long-read sequencing and demonstrated that a recombinant protein secreted by engineered *S. boulardii* in the intestine could change the microbiome. Our results provide valuable information for the development of therapeutics using engineered *S. boulardii* that changes the gut microbiome and host physiology.

## INTRODUCTION

Microbiome engineering, which aims to change the structure of the microbiome to induce health benefits, has received great attention in the food and pharmaceutical industries (1, 2). Microbiome engineering is mainly accomplished through five methods: probiotics, prebiotics, antibiotics, microbiota transplants, and bacteriophages, each with advantages and disadvantages (1). Probiotics affect the gut microbiome through nutritional competition, production of antimicrobial compounds, and modulation of host immunity, but predicting changes in an individual's microbiome is difficult (1, 2). Prebiotics induce the selective growth of desirable microorganisms, but rationally predicting changes in the microbiome and their consequences is challenging (1). The use of antibiotics can kill gut microbes, including many pathogens, but this leads to potentially undesirable changes, such as lowering the diversity of microorganisms in the intestine, inducing secondary infection by the growth of Clostridioides difficile, and creating antibiotic-resistant strains (1). Although intestinal microbiota transplantation is very effective in preventing the recurrence of *C. difficle* infection, its mechanism and consequences are poorly understood, so the FDA has warned against this (1, 2). Bacteriophages have the potential to kill certain strains in the intestine, but many technological advances and regulatory agreements are needed (1, 2).

To fine-tune the microbiome into its intended structure, many researchers focused on the use of genetically modified probiotics called “chassis” (1, 2). Several criteria determine suitability as a chassis (2): (i) the chassis or its metabolic activity must not have an adverse effect on humans (2, 3); (ii) genome editing tools for the chassis must be well-developed (4); (iii) the environmental resistance of the chassis must be sufficient to pass through the stomach (4); and (iv) the chassis must be capable of producing a sufficient amount of target molecules (proteins, metabolites, etc.) to induce the desired physiology (1, 2). Colonization of the chassis may be necessary for treating chronic diseases yet long-term colonization may cause side effects such as autoimmune diseases (2, 5). Thus, a short residence time is also desirable for a chassis.

*Saccharomyces boulardii* is a probiotic yeast that is recently being used as a host for microbiome engineering (6). As *S. boulardii* is a generally recognized safe strain, this yeast has been used for a long time as an over-the-counter drug for preventing and treating intestinal diseases (4). The technologies developed for genome editing of Saccharomyces cerevisiae can be applied almost as they are (6, 7). *S. boulardii* has been reported as highly resistant to environmental stresses such as acidity and body temperature compared to many other bacteria and yeast (4, 7, 8), and can secrete recombinant proteins in the intestine (9). As *S. boulardii* is thoroughly washed out of the intestine within 3 to 4 days after cessation of oral administration, the supply of recombinant protein can be easily controlled (6). Recent studies have demonstrated the potential of microbiome engineering by using genetically engineered *S. boulardii* (6, 10) or probiotic S. cerevisiae (11) to produce recombinant proteins in the intestine to treat inflammatory bowel disease or selectively inhibit C. difficile. In addition to these promising results, research to change the overall structure of the microbiome using *S. boulardii* will enable the development of new microbiome therapeutics based on probiotic yeast. Unfortunately, to date, no studies have investigated how *S. boulardii* administration alters the gut microbiome of normal animals. Additionally, no study has attempted to alter microbiome structure by manipulating genes in *S. boulardii* to secrete recombinant proteins in the intestine.

To use *S. boulardii* as a means of transforming a target microbiome into specific structures and inducing health benefits, two preliminary studies are essential. First, it is necessary to investigate changes in the gut microbiome caused by the administration of *S. boulardii* or recombinant proteins secreted by *S. boulardii*. However, in all the studies that investigated the gut microbiome changes following the administration of wild-type *S. boulardii* (12, 13), the experimental groups had inherent or induced intrinsic physiological changes, such as disease or treatment of tissue-damaging substances and antibiotics. Therefore, it was difficult to conclude that these changes were induced only by *S. boulardii* administration. In addition, prior studies performed short-read sequencing, which makes identifying the genus difficult due to its low resolution (13). As a result, most studies have reported changes only at the phylum or family level. More importantly, there have been no studies yet to change the gut microbiome by producing recombinant proteins through *S. boulardii*.

Second, quantifying the recombinant protein secreted by *S. boulardii* in the intestine is necessary. To date, several studies have reported the use of genetically engineered *S. boulardii* to produce recombinant proteins in the intestine (14) and validated the presence of recombinant proteins in the intestine through the expression of green fluorescent protein (GFP) (6) or mCherry (11). Along with the previous results, if the recombinant proteins secreted by *S. boulardii* can be quantified in the intestine, predicting the titer of therapeutic proteins and the resulting changes in host physiology can be possible.

In this study, to provide basic information for using *S. boulardii* as a chassis of microbiome engineering, we (i) investigated the changes in the gut microbiome resulting from *S. boulardii* administration in healthy mice; (ii) quantified intestinal levels of a recombinant protein produced by *S. boulardii* and investigated the effect of the recombinant protein on the gut microbiome of mice; and (iii) investigated the changes in fecal and serum metabolomes of mice administered with *S. boulardii* or recombinant protein-producing *S. boulardii*. To accomplish this, *S. boulardii* was genetically engineered to secrete human lysozyme through Cas9-based genome editing. For long-read sequencing with high accuracy at the genus and strain levels, PacBio long-read sequencing was used to analyze microbiome changes caused by *S. boulardii* with high accuracy (13).

## RESULTS

### Engineering of *S. boulardii* for secreting human lysozyme.

**FIG 1:**
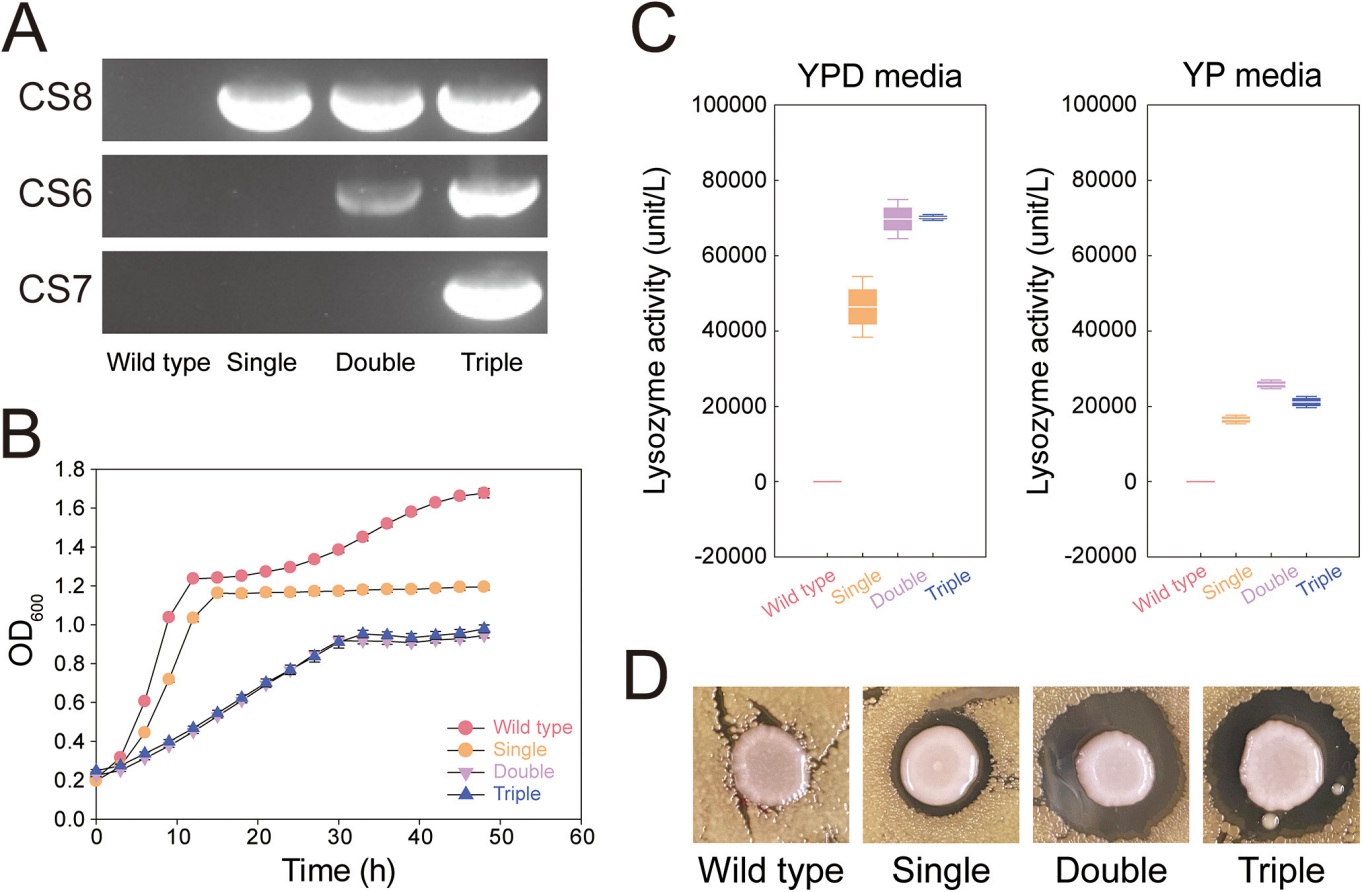
Construction of human lysozyme-secreting *S. boulardii*. (A) Insertion of a human lysozyme gene with chicken secretion signal into CS6, CS7, and CS8 sites for construction of single, double, and triple copy strains. (B) Growth curves of wild type and engineered *S. boulardii* in YPD medium at 37°C. (C) Lysozyme activity measured using lysis rates of *Micrococcus lysodeikticus*. (D) Lysozyme activity measured using a halo assay.

In a previous study, we integrated a human lysozyme gene with a chicken lysozyme secretion signal into an intergenic region (CS8 region) of the *S. boulardii* genome through Cas9-based genome editing (7). Metabolic activities of *S. boulardii* in the intestine might be restricted by highly abundant bacteria (6), and the secreted amounts of human lysozyme by the single-copy integrated *S. boulardii* may not be sufficient to induce changes in the gut microbiome. To increase the lysozyme-producing capability of *S. boulardii*, a human lysozyme expression cassette was integrated into the intergenic regions of CS6, CS7, and CS8 using Cas9-based genome editing (see Table S1 in the supplemental material). Engineered *S. boulardii* strains with single, double, and triple copies of the lysozyme expression cassette were constructed (Fig. 1A). Wild-type and engineered strains of *S. boulardii* were cultured in yeast extract-peptone-dextrose (YPD; 10 g/L yeast extract, 20 g/L peptone, and 20 g/L glucose) medium at 37°C and their growth rates were measured (Fig. 1B). Compared with the wild type, the engineered strains showed significantly lower growth rates and final cell densities after 10 h, but there were no significant differences between the double-copy strain and the triple-copy strain. The reduced growth rate with increased synthesis of heterologous proteins may be due to the accumulation of reactive oxygen species due to the increased demand for protein folding in the endoplasmic reticulum (15, 16). The lysozyme secretion ability of each strain was evaluated through the lysis of *Micrococcus lysodeikticus*, a lysozyme-sensitive Gram-positive strain (Fig. 1C and D). Lysozyme secretion in the double-copy strain was significantly higher than that in the single-copy strain and was like that in the triple-copy strain. Therefore, the double-copy strain with the highest lysozyme secretion and minimal genetic modifications was used for the mouse experiments.

### Administration of phosphate-buffered saline, *S. boulardii*, and lysozyme-secreting *S. boulardii* to mice.

**FIG 2:**
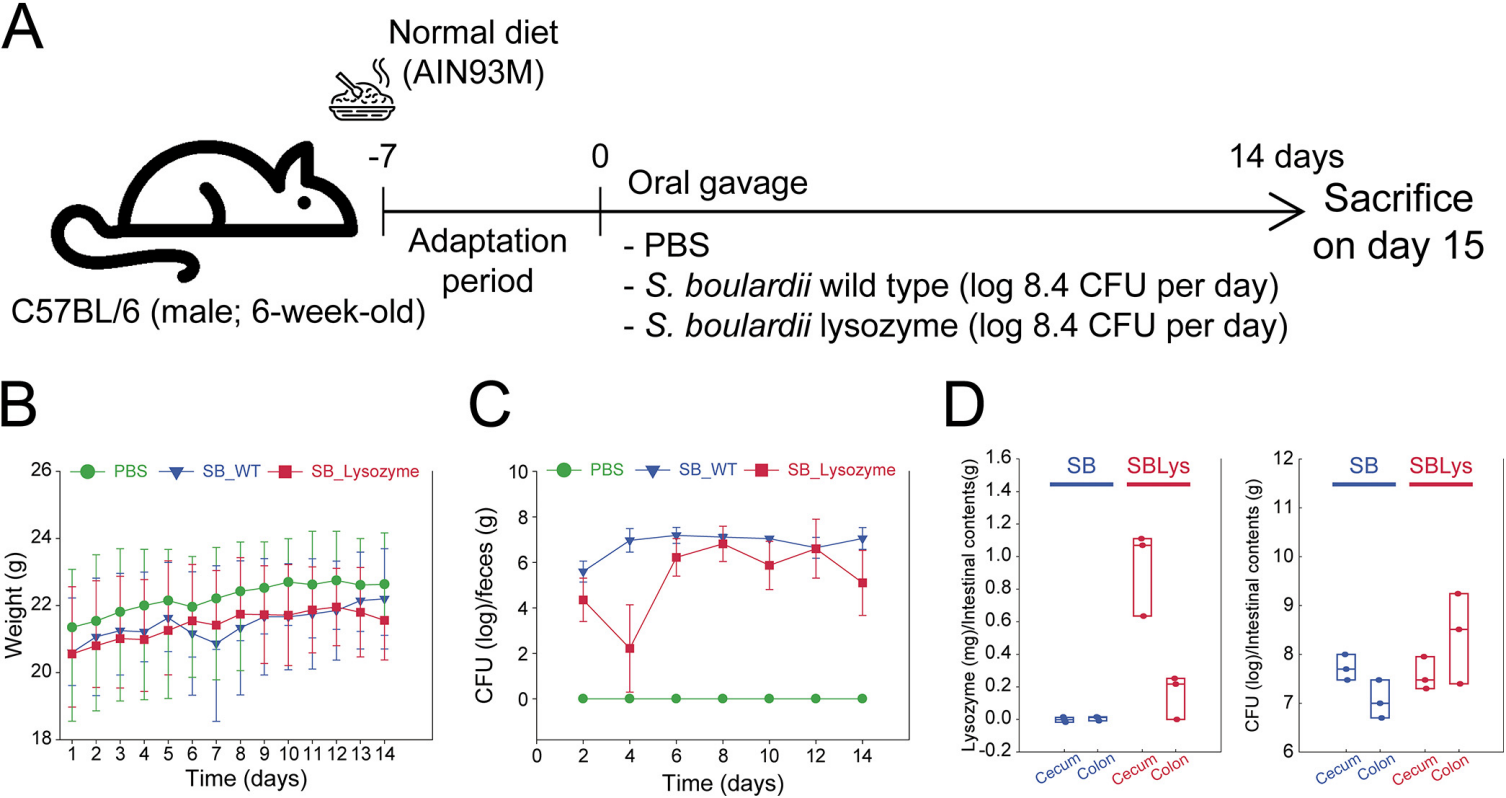
Overview of mouse experiments. (A) Schematic diagram of experiment. (B) Weight of mice during oral gavage period. (C) Yeast CFU of fecal samples. (D) Amounts of lysozyme and yeast CFU from the intestinal contents.

After acclimatization to the experimental environments for 1 week, 6-week-old mice were administered an oral gavage every day for 14 days and sacrificed on the 15th day (Fig. 2A). Treatments included *S. boulardii* wild-type (SB), *S. boulardii* secreting human lysozyme (SBLys), or vehicle control (phosphate-buffered saline [PBS]). In all cases, 100 μL was gavaged daily with PBS or PBS containing 3×10^8^ CFU of fresh-grown yeast cells. During the experimental period, there was no significant difference in the weights of the mice among the groups (Fig. 2B). Approximately 6 log yeast cells/g feces were observed from day 2 in SB and day 6 in SBLys (Fig. 2C). The amounts of lysozyme in the cecum and colon samples of mice sacrificed on day 15 were quantified by using a human lysozyme ELISA kit, but no human lysozyme was detected (data not shown). These results indicate that all residual heterologous proteins were excreted from the intestine 1 day after oral gavage was completed.

To quantify the lysozyme secreted by SBLys in the intestine, additional mice were purchased and daily oral gavage of SB or SBLys was performed for 5 days (*n* = 3 per group). Two hours after the last oral gavage, the mice were sacrificed, and duodenum, cecum, and colon contents were collected to quantify the amounts of lysozyme in the samples using a human lysozyme ELISA kit (Fig. 2D). As a result, 1.1 mg lysozyme/g cecal contents and 0.2 mg lysozyme/g colon contents were quantified in SBLys. Yeast CFU of SBLys were similar in the cecum and were significantly higher in the colon than in the SB.

### Overall changes in microbiome among the groups at phylum level.

**FIG 3:**
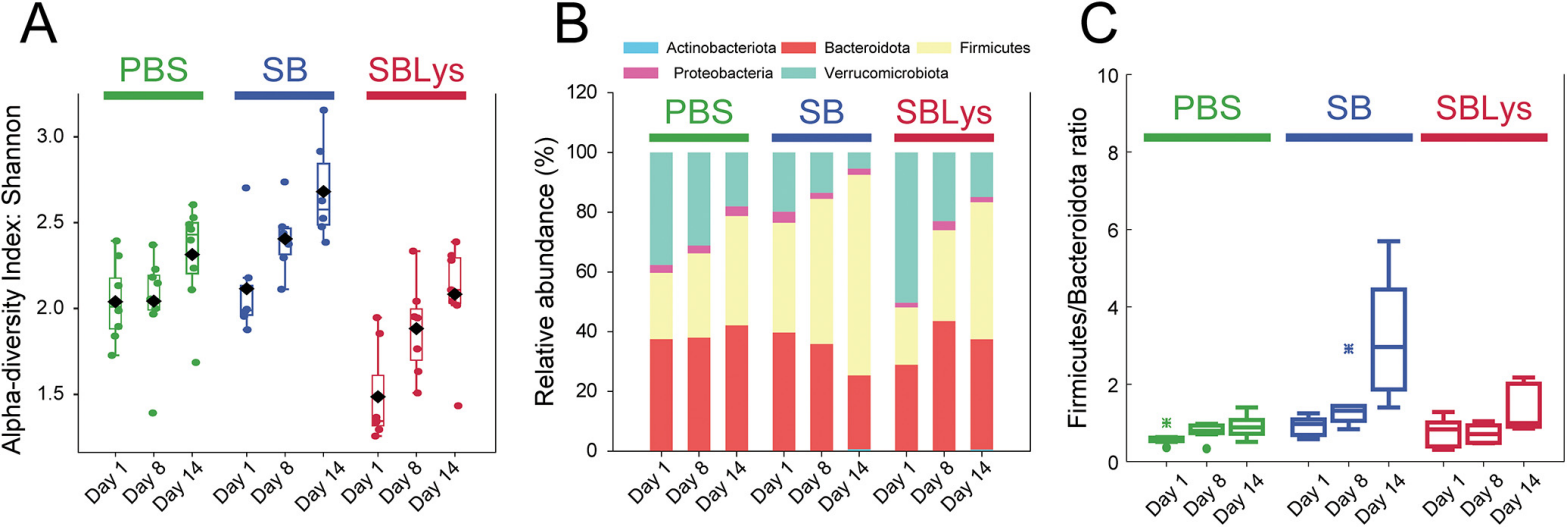
Overall changes in microbiome. (A) Alpha diversity. (B) Relative abundances of phyla. (C) *Firmicutes*/*Bacteroidota* ratio.

To understand changes in the gut microbiome following administration of SB and SBLys, 16S long-read sequencing using PacBio was performed using fecal samples on the 1st, 8th, and 14th days. A total of 1,331 amplicon sequence variants (ASVs) were identified (Table S2). Each ASVs may refer to a different microorganism. To investigate changes in the gut microbiome according to each treatment, alpha diversity, phylum composition, and *Firmicutes*/*Bacteroidota* ratio of all groups were compared (Fig. 3). The alpha-diversity of PBS group increased from 2.04 ± 0.23 on day 1 to 2.41 ± 0.30 on day 14 (Fig. 3A). The alpha-diversity of SB group increased from 2.12 ± 0.30 on day 1 to 2.68 ± 0.30 on day 14 (Fig. 3A). The alpha-diversity of SBLys group increased from 1.49 ± 0.29 on day 1 to 2.08 ± 0.32 on day 14 (Fig. 3A). The alpha diversity of all groups significantly increased with time, suggesting that the diversity of the gut microbiome in mice increased with the duration of the feeding experiments. Previous studies have reported that alpha diversity increases with age in mice (17, 18). Therefore, increased alpha diversity as the experiment continued and mice aged may have influenced our results. Changes in phylum composition over time were investigated for each treatment (Fig. 3B). In our results, the mouse microbiome was mostly composed of *Verrucomicrobiota*, *Bacteroidota*, and *Firmicutes*. *Verrucomicrobiota* decreased over time in all groups, but *Firmicutes* and *Bacteroidota* changed differently according to the treatment. In PBS group, *Firmicutes* and *Bacteroidota* increased in a balanced way with time, and the F/B ratios on day 1 and day 14 were 0.60 ± 0.19 and 0.91 ± 0.28, respectively (Fig. 3C). In contrast, in SB group, the ratio of *Firmicutes* to *Bacteroidota* significantly increased over time (Fig. 3B). The F/B ratio after administering SB was 0.91 ± 0.28 on day 1 and significantly increased to 3.22 ± 1.70 on day 14 (Fig. 3C). In SBLys group, the F/B ratio significantly increased from 0.74 ± 0.35 on day 1 to 1.30 ± 0.55 on day 14 (Fig. 3C). These results suggest that oral administration of SB or SBLys induced changes in the mouse microbiome by increasing the proportion of *Firmicutes*, and *Firmicutes* increased more after the administration of SB than SBLys.

### Changes in the gut microbiome by SB administration.

**FIG 4:**
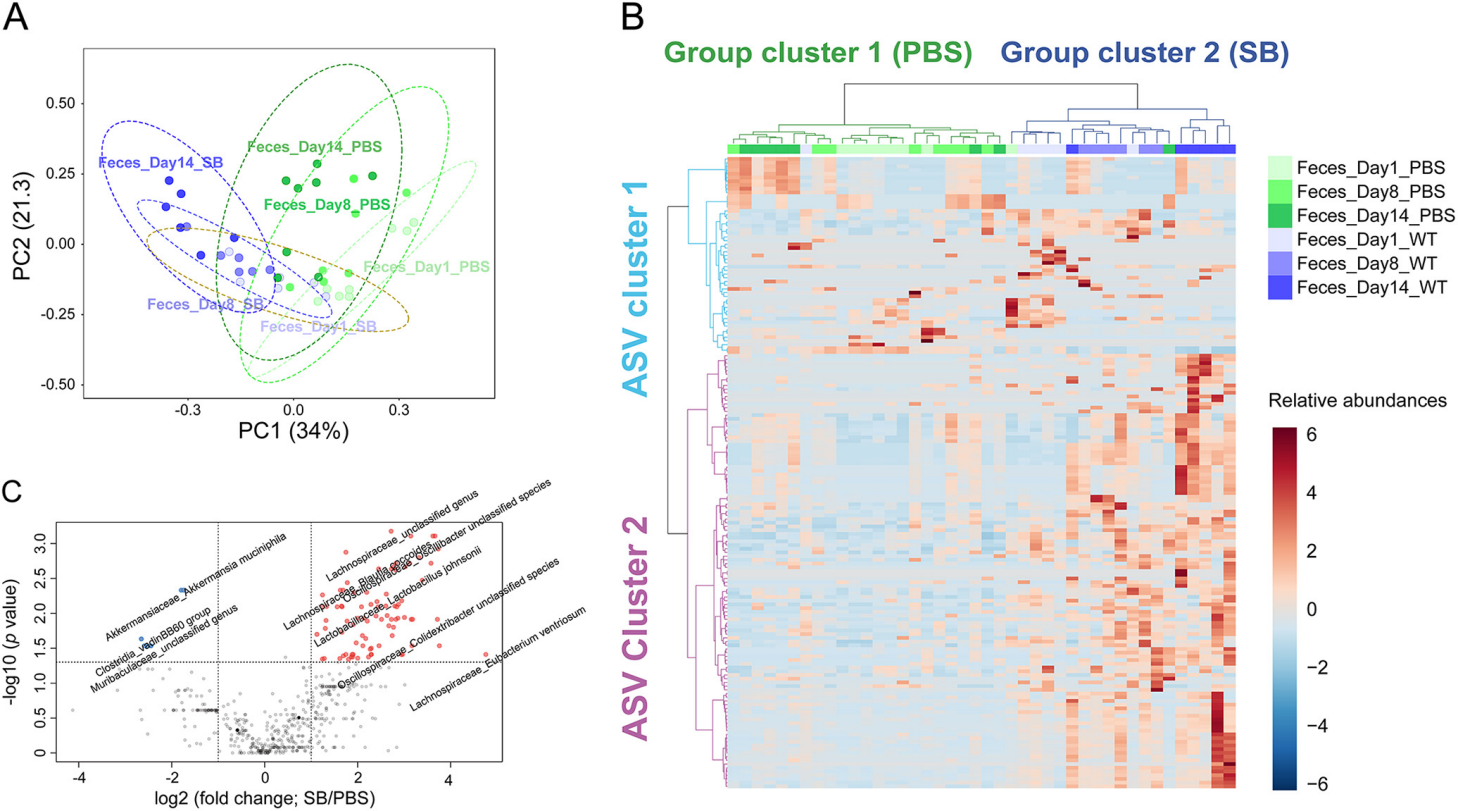
Comparison of fecal microbiomes between PBS and SB groups. (A) PCoA using 1,331 ASVs. (B) HCA using 1,331 ASVs. (C) Volcano plot for suggesting significantly changed ASVs between SB and PBS groups on day 14.

To investigate changes in the gut microbiome following SB administration, the fecal microbiome after PBS and SB administrations was compared over time using principal coordinates analysis (PCoA) and hierarchical clustering analysis (HCA). The PCoA model using 1,331 ASV showed that PBS and SB groups were completely distinct based on PC1 (Fig. 4A). The PC1 value of the PBS group decreased from 0.19 ± 0.11 on day 1 to 0.04 ± 0.09 on day 14. The PC1 value of SB decreased from –0.04 ± 0.11 on day 1 to –0.30 ± 0.07 on day 14. HCA was performed to investigate these changes in more detail (Fig. 4B). Fecal samples were divided into two groups, and the ASVs were divided into two ASV clusters. Cluster 1 mostly consisted of the PBS group, and cluster 2 mostly consisted of the SB group (Fig. 4B). ASV cluster 1 consisted of ASVs that were initially not abundant but increased in the PBS group over time or ASVs that did not change over time in each group. ASV cluster 2 consisted of ASVs that increased in the SB group over time, and the number of ASVs was much higher than that of ASVs in cluster 1. The PCoA and HCA results indicated that the microbiome compositions of the PBS and SB groups were similar each other on day 1 but completely different on day 14.

The fecal microbiomes from day 14 samples of PBS and SB were compared to reveal the individual microbes that were significantly altered after SB administration. Significantly altered microbes were selected based on two criteria (*P < *0.05, fold change < 2.0), and these were visualized as a volcano plot (Fig. 4C). Each ASV was labeled as a family_genus species (*n* = number of overlapping names). Among the 92 species that were significantly changed (Table S3), 85 ASVs increased significantly in SB, which were *Lachnospiraceae*_unclassified genus (36), *Lachnospiraceae*_NK4A136 group unclassified species (14), *Lachnospiraceae*_Blautia coccoides (6), *Oscillospiraceae*_unclassified genus (6), *Lachnospiraceae*_*Lachnospiraceae* bacterium NK4A136 group (3), *Oscillospiraceae*_*Colidextribacter* unclassified species (3), *Oscillospiraceae*_*Oscillibacter* unclassified species (3), *Lachnospiraceae*_*Eubacterium ventriosum* (2), *Lachnospiraceae*_*Lachnoclostridium* unclassified species (2), *Lachnospiraceae*_*Tuzzerella* unclassified species (2), *Lactobacillaceae*_Lactobacillus johnsonii (2), *Oscillospiraceae*_NK4A214 group unclassified species (2), UCG-010 unclassified genus (2), *Lachnospiraceae*_*Lachnospiraceae* bacterium A2 (1), and RF39_unclassified family (1). Among them, 82 ASVs were Clostridia, and the remaining three were *Bacilli*. Seven ASVs were significantly decreased in SB, including the *Muribaculaceae*_unclassified genus (4), *Akkermansiaceae*_*Akkermansia muciniphila* (2), and *Clostridia*_vadinBB60 group (1). The types of ASVs included four *Bacteroidia*, two *Verrucomicrobiae*, and one *Clostridia*.

### Changes in the gut microbiome by administering the human lysozyme-secreting *S. boulardii*.

**FIG 5:**
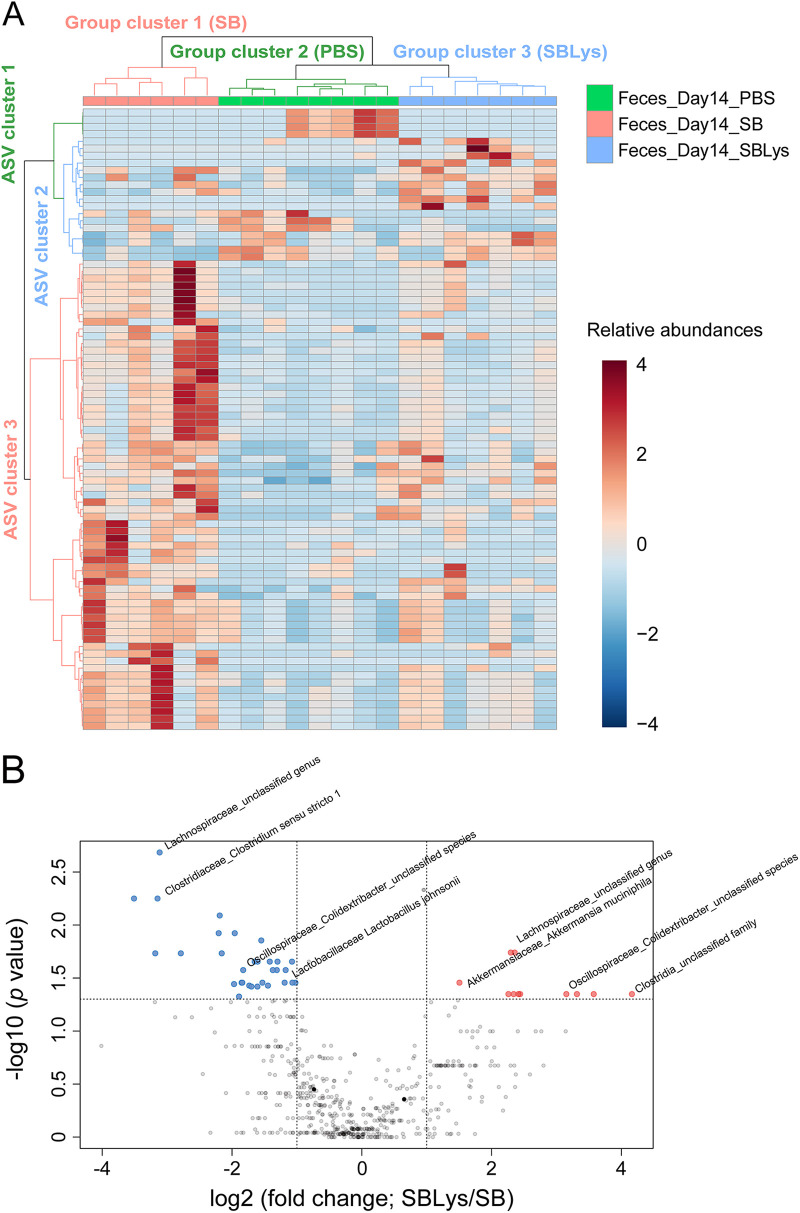
Comparison of fecal microbiome between SB and SBLys. (A) HCA using 86 significantly changed ASVs in comparison between PBS, SB, and SBLys. (B) Volcano plot for suggesting significantly changed ASVs between SB and SBLys on day 14.

To check whether human lysozyme secreted by *S. boulardii* caused further changes in the microbiome, fecal microbiomes from day 14 samples of the PBS, SB, and SBLys groups were compared. A total of 86 ASVs changed significantly depending on the treatment (*P < *0.05; Table S4). Three group clusters and three ASV clusters were obtained by performing HCA using 86 significantly changed ASVs (Fig. 5A). Cluster 1 consisted of the SB group, cluster 2 consisted of the PBS group, and cluster 3 consisted of the SBLys group. ASV cluster 1 consisted of ASVs of relatively high abundance in PBS, ASV cluster 2 consisted of ASVs of relatively high abundance in SBLys, and ASV cluster 3 consisted of ASVs of relatively high abundance in SB. ASV cluster 1 can be regarded as ASVs that are reduced by administering probiotic yeast (SB and SBLys). ASV cluster 2 can be considered ASVs increased by lysozyme secretion. ASV cluster 3 can be considered ASVs that could be increased by administration of probiotic yeast but maintained an abundance similar to that of the control group due to lysozyme sensitivity. The SBLys cluster differed from the PBS and SB groups, indicating that the secreted lysozyme changed the gut microbiome structure.

The fecal microbiomes the SB and SBLys groups on day 14 were compared to reveal the individual microbes that were significantly altered by lysozyme. A total of 42 ASVs were significantly changed (Table S5; Fig. 5B), and 11 ASVs, including *Lachnospiraceae*_unclassified genus (4), *Clostridia*_unclassified family (3), *Akkermansiaceae*_*Akkermansia muciniphila* (1), *Lachnospiraceae*_A2_unclassified species (1), *Lachnospiraceae*_*Lachnospiraceae* NK4A136 group (1), and *Oscillospiraceae*_*Colidextribacter* unclassified species (1) were significantly increased in SBLys compared to SB. A total of 31 ASVs were significantly lower in SBLys than in SB: *Lachnospiraceae*_unclassified genus (19), *Lachnospiraceae*_*Lachnospiraceae* NK4A136 group_ (3), *Lachnospiraceae*_NK4A136 group unclassified species (2), *Lachnospiraceae*_Blautia coccoides (2), *Lactobacillaceae*_Lactobacillus johnsonii (2), *Oscillospiraceae*_*Colidextribacter*_unclassified species (2), and *Clostridiaceae*_*Clostridium sensu stricto 1* (1).

### Changes in fecal and serum metabolome by SB and SBLys administration.

**FIG 6:**
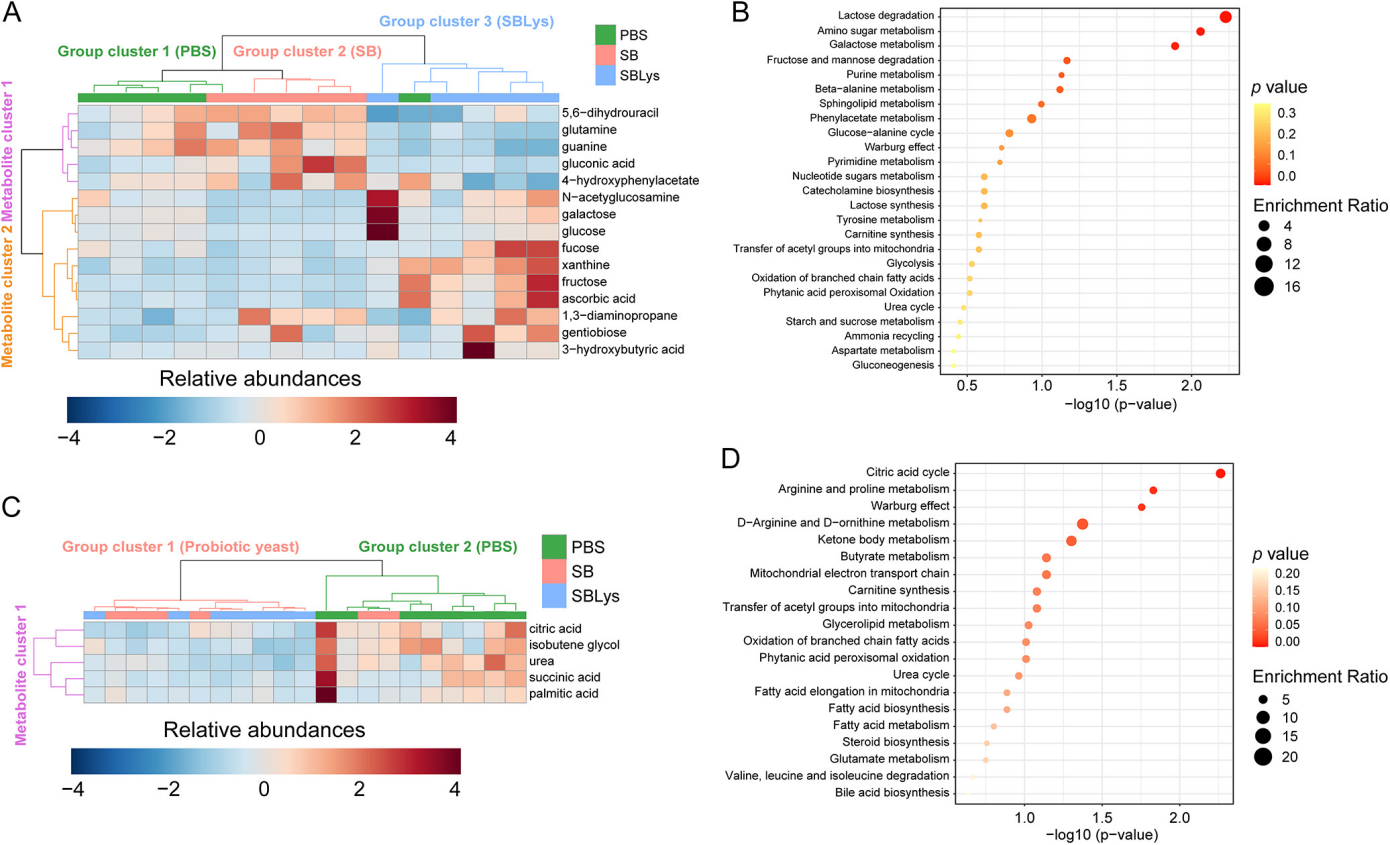
Changes in metabolome and biochemical metabolism. (A) HCA using 15 significantly changed fecal metabolites. (B) MSEA using 15 significantly changed fecal metabolites for suggesting significantly changed biochemical metabolism in gut microbiome. (C) HCA using 5 significantly changed serum metabolites. (D) MSEA using 5 significantly changed serum metabolites for suggesting significantly changed biochemical metabolism in host.

To investigate the changes in the metabolome induced by SB and SBLys administration, metabolites were extracted from feces and serum on day 14. A total of 79 fecal metabolites (Table S6) and 17 serum metabolites (Table S7) were identified using gas chromatography-mass spectrometry (GC-MS). The Kruskal-Wallis test was performed to identify individual metabolites that were significantly altered by each treatment. Fifteen fecal metabolites, including galactose, glucose, fructose, ascorbic acid, 5,6-dihydrouracil, xanthine, glutamine, gentiobiose, guanine, *N*-acetylglucosamine, gluconic acid, fucose, 3-hydroxybutyric acid, 4-hydroxyphenylacetate, and 1,3-diaminopropane, changed significantly (Table S6). HCA with 15 significantly altered fecal metabolites showed three group clusters and two metabolite clusters (Fig. 6A). Metabolite cluster 1 consisted of metabolites that were abundant in the SB group but less abundant in SBLys group. Metabolite cluster 2 is composed of metabolites abundant in the SBLys group and low in the PBS and SB groups, which are mainly saccharides, such as *N*-acetylglucosamine (GlcNac), galactose, glucose, fucose, fructose, and gentibiose. Metabolite enrichment analysis (MSEA) was performed to identify the highly affected biochemical metabolisms. Sugar metabolism, such as lactose degradation, amino sugar metabolism, galactose metabolism, fructose and mannose degradation, and nucleotide metabolism, such as purine metabolism and sphingolipid metabolism, were significantly altered (Fig. 6B). A total of five serum metabolites, urea, succinic acid, palmitic acid, isobutene glycol, and citric acid, changed significantly depending on the group (Table S7). HCA with five significantly changed serum metabolites showed two clusters, one composed of probiotic yeast (SB and SBLys) and the other composed of PBS (Fig. 6C). All metabolites were abundant in the PBS group, and low in the SB and SBLys groups. MSEA results showed that energy-related metabolic pathways, such as the citric acid cycle, Warburg effect, and mitochondrial electron transport chain, were significantly affected (Fig. 6D).

## DISCUSSION

To provide basic information for studies on treating diseases by changing the gut microbiome with *S. boulardii*, we engineered *S. boulardii* to secrete human lysozyme and investigated the changes in the microbiome and fecal and serum metabolomes induced by SB or SBLys administration in mice. The probiotic yeast *S. boulardii*, a potential chassis for microbiome engineering, is highly resistant to environmental stress, can secrete recombinant proteins with posttranslational modifications (PTMs) in the intestinal tract, has well-developed genetic tools, and is easily excreted from the intestine.

In this study, SB administration altered the structure of the gut microbiome by increasing the proportion of *Clostridia*. SB administration resulted in a unique gut microbiome structure composed of completely different types and abundances of ASVs compared to the PBS control (Fig. 4B). Specifically, the microbes were significantly increased by SB administration, including *Blautia*, *Tyzzerella*, NK4A136 group, NK4A214 group belonging to the *Lachnospiraceae* or *Oscillospiraceae* family, and L. johnsonii belonging to the *Lactobacillaceae* family. Previous studies have reported that administration of S. cerevisiae (19–21), which has a genome almost identical to that of SB (22), supplies nutrients such as mannan oligosaccharides, β-glucan, and amino acids to *Lachnospiraceae* and *Lactobacillus* to promote their growth. Similarly, SB administration appeared to increase the diversity of the bacterial gut microbiome (Fig. 3) by providing nutrients such as mannan oligosaccharides, β-glucan, and amino acids selectively to *Clostridia* such as *Lachnospiraceae*, and indirectly enhancing the growth of their symbiotic microorganisms. These results differ from those of previous studies that observed decreased *Firmicutes* and increased *Bacteroidota* in SB-administered mice (23, 24). We speculate that our results might differ from those of previous studies because they analyzed the gut microbiome of transgenic or liver-injured mice by short-read sequencing. However, we analyzed the gut microbiome of healthy mice using long-read sequencing. The increase in *Clostridia* and prevention of C. difficile infection (CDI) by SB administration may be related. For example, Clostridium scindens inhibits the growth of C. difficile by secreting tryptophan-derived antibiotics and converting the primary bile acids into the secondary bile acids (25). Further studies should investigate the presence of a gene for bile acid 7α-dehydration in *Clostridia* that was promoted by SB administration.

Human lysozyme secreted by engineered *S. boulardii* changes the gut microbiome structure and fecal metabolome. The lysozyme secreted by SBLys was detected in the cecum and colon (Fig. 2D) and resulted in a substantially different microbiome structure compared to those of the PBS control and SB (Fig. 5A). While the abundance of many microbes increased by SB administration remained similar to that in PBS in SBLys (ASV cluster 3; Fig. 5A), some microbes increased only in SBLys (ASV cluster 2; Fig. 5A). This may be a result of the growth of lysozyme-insensitive microorganisms when lysozyme secreted by SBLys in the intestine inhibits the growth of lysozyme-sensitive microorganisms. Interestingly, SBLys administration significantly increased the abundance of *A. muciniphila*, which was significantly reduced by SB administration. This result contrasted with the results of a previous study that reported a decrease in *A. muciniphila* after the administration of lysozyme to mice fed a high-fat diet (HFD) (26). This conflicting result may be due to the complex interaction of SB and lysozyme in the gut microbiome. Alternatively, this may be because a previous study significantly increased the abundance of *A. muciniphila* through HFD feeding in mice. In addition, administration of SBLys increased the concentrations of fecal sugars such as fucose and GlcNac. In the present study, lysozyme administration reduced the proportion of *Lactobacillus*, *Blautia*, and *Clostridium sensu stricto* (Fig. 5B), which are known to ferment fucose and GlcNac (27–30), and because their growth was inhibited by lysozyme, more sugars might have remained in the feces (Fig. 6A).

Administration of probiotic yeast may affect host energy metabolism and decrease blood urea and fructose levels either directly or indirectly by altering the gut microbiome. The administration of SB or SBLys affected host energy metabolism (Fig. 6D), including the citric acid cycle, Warburg effect, and mitochondrial electron transport chain, and induced a decrease in blood urea and fructose (*P = *0.07; Table S6) (Fig. 6C). Similar to our results, a previous study in which SB was administered to *db*/*db* mice showed a decrease in the markers of obesity and type 2 diabetes and an increase in *Lactobacillus* (23). This change may be due to the effects of probiotic yeast administration itself or the induced microbiome change. For example, *Blautia* (31), *Lachnospiraceae* (32), and Lactobacillus johnsonii (33), which were increased by probiotic yeast administration (Fig. 4C and 5A), are regarded as potential probiotics. Although the mechanisms by which these microbes induce human health benefits have not been completely elucidated, they are known to affect other gut microbes and hosts through competition for nutrients, production of short-chain fatty acids, and modulation of host immunity (31–33). Thus, selective growth of beneficial microorganisms such as *Blautia* and *Lactobacillus* by probiotic yeast administration may contribute to changes in energy metabolism and lowering blood urea and sugar levels (Fig. 6D, Table S7).

In this study, we identified changes in the gut microbiome following SB administration in healthy mice, unlike previous mouse studies on disease or treatment with tissue-damaging substances and antibiotics. We also demonstrated for the first time that a recombinant protein secreted by engineered SB in the gut could change the microbiome. Although our study used the latest technology, PacBio long-read sequencing, genus, and species information of many ASVs were not identified because of the lack of library information. As PacBio technology and libraries are being updated rapidly, further identification and application will be soon possible using raw data. In addition, future research needs to increase the amount of human lysozyme secreted in the intestine through the expression of a gene that helps protein secretion in yeast and investigate its effect on the microbiome. Our findings provide essential information for the use of SB as a chassis for microbiome engineering and can be used to develop functional foods and therapeutics.

## MATERIALS AND METHODS

### Strains and plasmid construction.

Escherichia coli Top10 was used for plasmid propagation. E. coli cells were cultivated in Luria-Bertani medium (LB; 5 g/L yeast extract, 10 g/L tryptone, and 10 g/L NaCl) at 37°C, and ampicillin (100 g/L) was added for selection. *S. boulardii* ATCC MYA-796 was used in this study. YPD was used as medium for yeast culture. To integrate human lysozyme genes with a chicken lysozyme signal sequence (cHLY) into the genome of *S. boulardii* for stable expression, guide plasmids p42H-CS6, p42K-CS7, p42K-CS8, and plasmid p426-pGPD-cHLY were used (Table S1). A DNA fragment containing pGPD-cHLY was amplified from p426-pGPD-cHLY using primers CS6-IU and CS6-ID, CS7-IU and CS7-ID, and CS8-IU and CS8-ID (Table S1) and used as donor DNA for CRISPR-Cas9-based genomic integration. CS6-CKU and CS6-CKD, CS7-CKU and CS7-CKD, and CS8-CKU and CS8-CKD primers (Table S1) were used for diagnostic PCR for the correct integration of cHLY. Transformation of yeast cells was carried out with the polyethylene glycol (PEG)-LiAc method (7). Then, 3 μg of DNA was used for Cas9 or guide RNA plasmid transformation and 15 μg of donor DNA was used for homologous recombination. Genomic integration was confirmed using yeast colony PCR. Yeast strains transformed with plasmids containing the antibiotic markers were propagated on YPD agar plates supplemented with the corresponding antibiotics.

### Quantification of human lysozyme.

The amount of human lysozyme was measured using a human lysozyme enzyme-linked immunosorbent assay (ELISA) kit (ab267798, Abcam). Briefly, 50 μL of the standard or sample was added to 96 wells. Then, 50 μL of the antibody cocktail was added to all wells. The 96 wells containing the mixture were incubated at 30°C for 1 h and then washed three times with 350 μL of wash buffer. Next, 100 μL of 3,3′,5,5′-tetramethylbenzidine development solution was added to each well and incubated for 15 min at room temperature. Finally, 100 μL of stop solution was added to each well and the optical density (OD) was measured at 450 and 600 nm. The lysozyme solution included in the human lysozyme ELISA kit was diluted to 2,000, 1,000, 500, 250, 125, 62.5, and 31.25 ng/mL and used as a standard (*n* = 2).

For the quantification of human lysozyme from yeast culture, wild-type and engineered strains of *S. boulardii* were precultured in 5 mL YPD media in 15 mL culture tubes for 24 h. The precultured cells were centrifuged at 21,130 × *g* for 5 min at 4°C and washed with distilled water. The washing process was repeated three times. The washed cells were inoculated (initial OD=0.2) into 5 mL YPD in 15 mL culture tubes for 72 h. After centrifugation at 21,130 × *g* for 5 min at 4°C, 50 μL of the supernatant was used for the quantification of human lysozyme.

For the quantification of human lysozyme from intestinal contents, 50 mg of cecal and colon contents was mixed with 500 μL of PBS buffer. Intestinal contents were broken down into small particles using sterile loops. The mixture of intestinal contents and PBS was vortexed vigorously for 10 min. The mixture was then centrifuged at 21,130 × *g* for 20 min at 4°C. Fifty μL of the supernatant was used for the quantification of human lysozyme.

### Mouse experiments.

Animal use for Project Protocol 21028 was approved on 5 March 2021, by the Illinois Institutional Animal Care and Use Committee according to the National Institutes of Health guidelines. Mice were purchased from the Jackson Laboratory and maintained at the animal facility of the Carl R. Woese Genomic Biology Institute at the University of Illinois at Urbana-Champaign. Six-week-old C57BL/6 male mice were maintained under a 12 h light/dark cycle in the feeding environment. The mice were acclimated to an AIN93M pellet diet (F3155, Bio-Serv) for 1 week. The mice were housed in groups of three. The cages were changed daily, each mouse was weighed, and the fecal samples were collected.

### Measurement of intestinal human lysozyme in mice.

To quantify human lysozyme secreted by the engineered *S. boulardii* from the intestine, three mice were used in each group (SB and SBLys). PBS buffer (100 μL PBS buffer containing SB or SBLys [3×10^8^ CFU]) was orally administered to each mouse daily for 5 days. Two hours after the last oral gavage, the mice were sacrificed, and the intestinal contents were collected.

### Investigation of the gut microbiome in mice.

Eight mice were used in each group to examine changes in the microbiome following SB or SBlys administration. One hundred μL of PBS or PBS containing yeast cells (3×10^8^ CFU) were orally administered to each mouse daily for 14 days. One day after the last oral gavage, the mice were sacrificed, and serum and intestinal contents were collected. During oral gavage, two mice from SB and one mouse from SBLys were sacrificed because of significant weight loss.

### Extraction of genomic DNA from fecal samples.

Genomic DNA was extracted from fecal samples using the QIAamp Fast DNA stool minikit (51604, Qiagen Sciences). Feces (50 mg) were mixed with 1 mL of InhibitEX buffer in a 2-mL microcentrifuge tube on ice. The feces were broken down into small particles using sterile loops. The tube containing the mixture was vortexed for 5 min and then heated for 5 min at 70°C. The tubes were vortexed again for 1 min and centrifuged at 21,130 × *g* for 5 min at 4°C. The supernatant (600 μL) was transferred into a new 2-mL microcentrifuge tube containing 25 μL proteinase K. Buffer AL (600 μL) was added to the mixture. Then, mixture was incubated at 70°C for 10 min and then mixed with 600 μL pure ethanol. The mixture was vortexed for 10 s and then transferred to a QIAamp spin column. The column was centrifuged at 21,130 × *g* for 1 min at 4°C. After all lysates were loaded onto the QIAamp spin column, 500 μL of buffer AW1 was added and centrifuged at 21,130 × *g* for 1 min at 4°C. Next, 500 μL of buffer AW2 was added and centrifuged at 21,130 × *g* for 3 min at 4°C. The spin column was placed in a new 2-mL collection tube and centrifuged at 21,130 × *g* for 3 min at 4°C. Finally, the spin column was transferred into a new 1.5-mL microcentrifuge tube, and 200 μL buffer ATE was added. After incubation for 1 min at room temperature, the column was centrifuged at 21,130 × *g* for 1 min at 4°C to elute DNA. The extracted genomic DNA was stored at −80**°**C until further analysis.

### Yeast CFU calculation.

To calculate viable yeast cells, 20 mg feces or cecal and colon contents were mixed with 1 mL PBS buffer. The feces were broken down into small particles using sterile loops and vortexed for 5 min. The mixture was diluted with PBS until an appropriate level of CFU was reached. The diluted mixture (200 μL) was spread onto Sabouraud dextrose agar containing chloramphenicol (50 mg/L). To express the case where there were no colonies on a log scale, 1 was added to all result values and transformed into a log.

### 16S rRNA gene long-read sequencing.

Library construction and sequencing in PacBio Sequel IIe were performed at the Roy J. Carver Biotechnology Center, University of Illinois at Urbana-Champaign. The 16S amplicons were generated using barcoded full-length 16S primers from Pacific Biosciences following their protocol. The forward and reverse 16S sequences were 5′-AGRGTTYGATYMTGGCTCAG-3′ and 5′-RGYTACCTTGTTACGACTT-3′, respectively. The individually barcoded amplicons were pooled and converted to a library using the SMRTBell Express template prep kit 2.0. The library was quantitated using Qubit and run on a fragment analyzer (Agilent) to confirm the presence of DNA fragments of the expected size. The library was sequenced on an SMRTcell 8M on PacBio Sequel IIe with a movie time of 12 h. Circular consensus analysis was performed using SMRTLink V10.1 with the following parameters: ccs min-passes 3 ***--***min-rq 0.999. Demultiplexing was performed using the lima software with the following parameters: ***--***ccs ***--***different ***--***split-bam-named. 16S rRNA data were preprocessed using the DADA2 pipeline (version 1.20.0) using default parameters (34). The Silva library (version 138.1) was used for taxonomic identification of bacteria (35, 36).

### Analysis of fecal and serum metabolites by using GC-MS.

To extract metabolites from feces, approximately 50 mg of fecal samples collected on day 14 were weighed and placed in 1.7-mL microcentrifuge tubes. After adding distilled water to a final concentration of 300 mg/mL, feces were broken down into small particles using sterile loops. The mixture was vigorously vortexed for 10 min and centrifuged at 21,130 × *g* for 20 min at 4°C. Next, 10 μL of the supernatant was transferred to a new 1.7-mL microcentrifuge tube and dried under reduced pressure in a Speed-Vac until all the solvents evaporated. To extract metabolites from the serum, 100 μL of serum was mixed with 900 μL of pure methanol and vortexed vigorously for 10 min. After centrifugation at 21,130 × *g* for 20 min at 4°C, 500 μL of the supernatant was transferred to a new 1.7-mL microcentrifuge tube and dried under reduced pressure on a Speed-Vac until all solvents evaporated.

To identify and quantify metabolites using GC-MS, methoximation and silylation were performed. For methoximation, 10 μL of 40 mg/mL methoxyamine hydrochloride (226904, Sigma-Aldrich) in pyridine (360570, Sigma-Aldrich) was added to the tubes containing the metabolites, and the mixture was incubated at 30°C for 90 min. For silylation, 50 μL of *N*-methyl-*N*-trimethylsilyl-trifluoroacetamide (69479, Sigma-Aldrich) was added to the mixture and incubated at 37°C for 30 min.

An Agilent 7890A GC coupled with a quadrupole MS5975C system (Agilent Technologies, Santa Clara, CA, USA) was used for metabolite analysis. A 1.0-μL aliquot of the derivatized sample was injected into the GC in splitless mode. The metabolites were separated on an RTX-5Sil MS column (30 m length, 0.25 mm inner diameter, and 0.25 μm film thickness; Restek) with an additional 10-m guard column. The initial oven temperature was set to 50°C for 1 min, ramped to 330°C at a rate of 20°C/min, and held at 330°C for 5 min. The mass spectra were recorded over a mass range of 85 to 550 *m/z*. The sample was ionized via electron impact at 70 eV. For the processing of the GC-MS data, MS dial (version 4.24) was used (37). The latest All records with the Fiehn RI library (38, 39) provided by MS-DIAL were used to identify metabolites by matching the mass spectra of the peaks.

### Statistical analyses.

For statistical analyses of the microbiome data, MicrobiomeAnalyst (https://www.microbiomeanalyst.ca/MicrobiomeAnalyst) were used (40). In each comparison, only ASVs with ≥2 counts were used for data analysis. A low-count filter and a low-variance filter were not used. The sum of the ASVs for each sample was scaled to 100. The following criteria were used for the calculation of alpha diversity (original data, Shannon diversity, and Mann-Whitney/Kruskal-Wallis). The following criteria were used for PCoA (Bray-Curtis Index, feature level, and permutational MANOVA). Ward clustering was used for the HCA Euclidean distance; Ward clustering. Correlations between ASVs were calculated using Spearman’s rank function of the SciPy package in Python (version 3.8). MetaboAnalyst (https://www.metaboanalyst.ca/; version 5.0) was used for statistical analyses of the metabolome data (41). Autoscaling was used for metabolite normalization.

### Data availability.

The data that support the findings of this study are openly available in Mendeley Data at https://doi.org/10.17632/khtyyfrsxw.1.
