# Supplementary material for: Microbiome Engineering Using Probiotic Yeast: Saccharomyces boulardii and the Secreted Human Lysozyme Lead to Changes in the Gut Microbiome and Metabolome of Mice
Source: Microbiol Spectr. 2023 Jul 12;11(4):e00780-23. doi: 10.1128/spectrum.00780-23 (PMC10433837; doi:10.1128/spectrum.00780-23)
Supplement: Supplemental file 1 — Table S1. Download spectrum.00780-23-s0001.docx, DOCX file, 0.02 MB [file spectrum.00780-23-s0001.docx]

**Table S1. Primers, plasmids, and strains used in this study**

| **Primers** | **Primer sequence** | **Source and/or reference(s)** |
| --- | --- | --- |
| CS6-IU | aacctcgaggagaagtttttttacccctctccacagatccaggaaacagctatgaccatg | (1) |
| CS6-ID | taattaggtagaccgggtagatttttccgtaaccttggtgtctgtaaaacgacggccagt | (1) |
| CS6-CKU | gtctgccgaaattctgtg | (1) |
| CS6-CKD | cggtcagaaagggaaatg | (1) |
| CS7-IU | tcgaaatttgttagatgccccgaataaatagtaactttatttagggaaagaattaaccctcactaaaggga | In this study |
| CS7-ID | gtgatggcgaaatgggtcttttactaagtacatgctaaaatatggcgtgagtaatacgactcactatagggc | In this study |
| CS7-CKU | gtattgtgacgcgttttcc | In this study |
| CS7-CKD | cctcccaactacatcttga | In this study |
| CS8-IU | caaaattacctacggtaattagtgaaaggccaaaatctaatgttacaataaattaacc ctcactaaaggga | (2) |
| CS8-ID | gaccgttcccttgtgttgtaccagtggtagggttcttctcggtagcttctgtaatac gactcactatagggc | (2) |
| CS8-CKU | agtggaacatagaagggg | (2) |
| CS8-CKD | taagcagcccagtgaac | (2) |
| **Plasmids** |  |  |
| p426-pGPD-cHLY | pRS426-pTDH3-cHLY-tCYC1 | (2) |
| Cas9-NAT | p414-TEF1p-Cas9-CYC1t-NAT1 | Addgene plasmid 64329; (3) |
| **Strains** |  |  |
| *S. boulardii ATCC MYA-796* |  | ATCC |
| S.b (Lysozyme; single copy) | CS8-cHLY | (2) |
| S.b (Lysozyme; double copy) | CS8-cHLY, CS6-cHLY | This study |
| S.b (Lysozyme; triple copy) | CS8-cHLY, CS6-cHLY, CS7-cHLY | This study |

1. Kwak S, Kim SR, Xu H, Zhang G-C, Lane S, Kim H, Jin Y-S. 2017. Enhanced isoprenoid production from xylose by engineered *Saccharomyces cerevisiae*. Biotechnol Bioeng 114:2581–2591.

2. Liu J-J, Kong II, Zhang G-C, Jayakody LN, Kim H, Xia P-F, Kwak S, Sung BH, Sohn J-H, Walukiewicz HE, Rao CV, Jin Y-S. 2016. Metabolic engineering of probiotic *Saccharomyces boulardii*. Appl Environ Microbiol 82:2280–2287.

3. Zhang G-C, Kong II, Kim H, Liu J-J, Cate JHD, Jin Y-S. 2014. Construction of a quadruple auxotrophic mutant of an industrial polyploid *Saccharomyces cerevisiae* strain by using RNA-guided Cas9 nuclease. Appl Environ Microbiol 80:7694–7701.
